# Myofascial Pain Syndrome: A Nociceptive Condition Comorbid with Neuropathic or Nociplastic Pain

**DOI:** 10.3390/life13030694

**Published:** 2023-03-03

**Authors:** César Fernández-de-las-Peñas, Jo Nijs, Barbara Cagnie, Robert D. Gerwin, Gustavo Plaza-Manzano, Juan A. Valera-Calero, Lars Arendt-Nielsen

**Affiliations:** 1 Department of Physical Therapy, Occupational Therapy, Physical Medicine and Rehabilitation, Universidad Rey Juan Carlos (URJC), 28922 Madrid, Spain; 2 Center for Neuroplasticity and Pain (CNAP), Sensory Motor Interaction (SMI), Department of Health Science and Technology, Faculty of Medicine, Aalborg University, 9220 Aalborg, Denmark; 3 Pain in Motion Research Group (PAIN), Department of Physiotherapy, Human Physiology and Anatomy, Faculty of Physical Education & Physiotherapy, Vrije Universiteit Brussel, 1050 Brussel, Belgium; 4 Chronic Pain Rehabilitation, Department of Physical Medicine and Physiotherapy, University Hospital Brussels, 1090 Jette, Belgium; 5 Department of Health and Rehabilitation, Unit of Physiotherapy, Institute of Neuroscience and Physiology, Sahlgrenska Academy, University of Gothenburg, 405 30 Gothenburg, Sweden; 6 Department of Rehabilitation Sciences, Ghent University, 9000 Ghent, Belgium; 7 Department of Neurology, School of Medicine, Johns Hopkins University, Baltimore, MD 21205, USA; 8 Department of Radiology, Rehabilitation and Physiotherapy, Complutense University of Madrid, 28040 Madrid, Spain; 9 Grupo InPhysio, Instituto de Investigación Sanitaria del Hospital Clínico San Carlos (IdISSC), 28040 Madrid, Spain; 10 Department of Medical Gastroenterology, Mech-Sense, Aalborg University Hospital, 9000 Aalborg, Denmark

**Keywords:** myofascial pain, trigger points, nociceptive pain, nociplastic pain, musculoskeletal pain, precision medicine, peripheral sensitization, central sensitization

## Abstract

Myofascial pain syndrome is featured by the presence of myofascial trigger points (TrPs). Whether TrPs are primary or secondary phenomena or if they relate to central or peripheral nervous system disorders is controversial. Referred pain, a cardinal sign of TrPs, is a central phenomenon driven by peripheral input. In 2021, the International Association for the Study of Pain (IASP) proposed a clinical criteria and grading system for classifying patients with pain on nociceptive, neuropathic, or nociplastic phenotypes. Myofascial TrP pain has been traditionally categorized as a nociceptive phenotype; however, increasing evidence supports that this condition could be present in patients with predominantly nociplastic pain, particularly when it is associated with an underlying medical condition. The clinical response of some therapeutic approaches for managing TrPs remains unclear. Accordingly, the ability to classify myofascial TrP pain into one of these phenotypes would likely be critical for producing more successful clinical treatment outcomes by a precision medicine approach. This consensus paper presents evidence supporting the possibility of subgrouping individuals with myofascial TrP pain into nociceptive, nociplastic, or mixed-type phenotype. It is concluded that myofascial pain caused by TrPs is primarily a nociceptive pain condition, is unlikely to be classified as neuropathic or nociplastic, but can be present in patients with predominantly neuropathic or nociplastic pain. In the latter cases, management of the predominant central pain problem should be a major treatment goal, but the peripheral drive from TrPs should not be ignored, since TrP treatment has been shown to reduce sensitization-associated symptomatology in nociplastic pain conditions, e.g., fibromyalgia.

## 1. Introduction

Myofascial pain syndrome is a condition characterized by the presence of myofascial trigger points (TrPs) and is a commonly overlooked and ignored cause of musculoskeletal pain. The presence of TrPs can be present as a primary dysfunction (i.e., mainly responsible of the pain of the patient) or can be present as a secondary phenomenon associated with other underlying medical diagnoses (i.e., a perpetuating factor of the symptoms) [1]. The most accepted definition for a TrP is “hypersensitive spot located into a taut band of a skeletal muscle that is painful upon stimulation of the muscle, which usually features a referred pain pattern and associated phenomena” [2]. This definition has been supported by a Delphi study where experts proposed the following TrP diagnostic criteria: 1, presence of a hypersensitive spot in the taut band, and 2, referred pain sensation elicited by stimulation of the point [3].

Trigger points can be clinically classified as active or latent depending on their relevance to the patient’s clinical presentation. A TrP is considered clinically active when the local and referred pain elicited by its stimulation reproduces, totally or partially, any symptom experienced by the patient, and the patient recognizes the symptom as a usual experience [2,3]. A TrP is considered clinically latent when the local and referred pain elicited by its stimulation does not reproduce any symptom experienced by the patient and the patient does not recognize that symptom as a usual/familiar experience [2,3]. Nevertheless, current literature on TrP pain is highly varied in its use of diagnostic criteria and no gold standard exists for its assessment. A review of diagnostic criteria of clinical trials investigating myofascial pain interventions further concluded that diagnostic criteria were heterogenous, since 23 combinations of diagnostic criteria were identified [4]. In that review, the most frequently used combination in 22% of the trials was spot tenderness, referred pain, and local twitch response, meaning that less than 25% of current clinical trials on myofascial TrP treatment have used the recommended diagnostic criteria [2,3].

Articles about myofascial trigger points have increased in number significantly over the past decade. Although an animal model [5] of myofascial pain has been purported to be developed, and two preliminary studies [6,7] have identified the presence of contracted sarcomeres in TrP areas in humans, there remains a vigorous debate about the etiology of TrPs. No objective biomarker that would be both sensitive and specific for myofascial pain has been found [8,9]. Data support a bidirectional relationship where TrPs may be a primary cause of nociception, and accordingly, peripheral and central sensitization, but also that TrPs can occur as a result of central sensitization processes and, in such a case, can be considered a secondary phenomenon [10]. Given these considerations, especially that TrPs can develop as a result of central sensitization, and can persist because of central sensitization, it is necessary to bring the nomenclature of myofascial pain up to date.

Three main pain phenotypes are currently being used to describe painful conditions: nociceptive, neuropathic, and nociplastic [11]. An additional phenotype would be the mixed type. In the past, TrPs have been considered as nociceptive pain. However, increasing evidence suggests that in some individuals, TrPs could also be present concurrently with predominantly neuropathic or nociplastic pain, a situation which could create difficulty in developing a treatment plan for patients.

Nociplastic pain is defined by the International Association for the Study of Pain (IASP) as “pain that arises from altered nociception despite no clear evidence of actual or threatened tissue damage causing the activation of peripheral nociceptors or evidence for disease or lesion of the somatosensory system causing the pain” [11]. Though it is well-established in current literature, this definition has raised questions [12]. First, discrimination between the pain phenotypes can be clinically challenging, since patients may fit into more than one pain phenotype (e.g., mixed type), and since one type (e.g., nociceptive) does not exclude another (e.g., nociplastic) [13]. Second, the clinical determination of altered nociceptive pain processing (sensitization) required for classifying a patient as having a nociplastic phenotype can be challenging, since no gold standard exists for determining whether a patient experiences a heightened pain response. In 2021, the IASP proposed the first set of clinical criteria and a system for identifying these three pain phenotypes [14]. The criteria are comprehensive, robust, properly developed, and with a high potential to be used by clinicians [15]. Although myofascial TrP pain could be considered primarily a nociceptive pain condition, fulfilling the definition of chronic primary musculoskeletal pain [16], in some patients the IASP nociplastic criteria could also be fulfilled, thereby classifying the pain phenotype as also nociplastic, leading the clinician to adapt treatment accordingly.

Identification of patients with a nociplastic pain phenotype has the potential to improve precision pain medicine practices in musculoskeletal pain conditions, although no randomized clinical trial has yet proven this hypothesis [17]. In the current consensus paper, an international group of experts in myofascial pain propose a clinical rationale for the application of the 2021 IASP clinical criteria to classify patients with myofascial TrP pain as predominantly nociceptive, neuropathic, or nociplastic. A proper classification of pain phenotypes is important because nociplastic pain is more challenging to treat, and because the treatment paradigms are more complicated than those for treatment of nociceptive pain. In fact, some treatment approaches with a high probability of success in nociceptive pain could be ineffective or even exacerbate the symptoms in patients with nociplastic pain.

## 2. Phenotyping Myofascial Trigger Point Pain

### 2.1. Nociceptive Pain Phenotype

Nociceptive pain is defined as pain attributable to the activation of the peripheral receptive terminals of primary afferent neurons in response to noxious chemicals and mechanical or thermal stimuli [18], and clinically, the pain response is proportional to the nociceptive input [19]. Myofascial TrP pain seems to be mainly considered a nociceptive pain. This classification is based on muscle pain being associated with the activation of muscle nociceptors by a variety of endogenous substances. Different studies have identified high concentrations of bradykinin (BK), calcitonin gene-related peptide (CGRP), nerve grown factor (NGF), interleukins 1β, IL-6, and IL-8, substance P, serotonin (5-HT), norepinephrine, and tumor necrosis factor-α (TNF-α) in active TrPs when compared with latent TrPs or control points [20,21,22]. However, some studies reported the presence of both nociceptive (hyperalgesia) and non-nociceptive (allodynia) sensitivity at the TrP area, indicating an overlap or mixed-pain phenotype [23,24,25].

The development or activation of TrPs can result from a variety of factors affecting the muscle tissue, e.g., repetitive muscle overuse, acute muscle overload, or repetitive minor muscle trauma [26]. Trigger points are located in a taut band, i.e., discrete bands of hardened muscle fibers, the nature of which is currently in dispute (contracted fiber bands or neurogenic edema). In fact, it has been found that taut muscle bands harboring TrPs exhibit greater stiffness than normal muscle [27], reduced vibration amplitude [28], and higher peak systolic velocity and negative diastolic velocity [29] when compared with normal muscle sites. More recently, evidence of overall increased stiffness in the entire muscle and not just in the taut band has been identified [30]. The integrated hypothesis postulates that TrPs represent a primary dysfunction of the motor endplate that results in sensitized peripheral nerve endings (nociceptive pain phenotype) [31].

As such, TrPs, particularly active TrPs, can act as sources of persistent or long-lasting peripheral nociceptive input, independent of tissue damage [32]. Obviously, active TrPs represent a focus of peripheral nociception, but it has been also observed that latent TrPs contribute to the process of nociception, but without reaching the thresholds to activate ascending pain pathways from the dorsal horn to the brain [33,34]. Mense proposed that latent TrPs refer pain to distant sites when usually ineffective (silent) synaptic connections in the dorsal horn are sensitized and become effective or active [35]. Since anesthetization of the referred pain area results in pain reduction, peripheral processes appear to contribute to referred pain (a central phenomenon) [36]. Ambite-Quesada et al. showed that latent TrPs were associated with mechanical, but not thermal, pain hyperalgesia in the affected area, but not in the referred pain area, suggesting that TrPs, at least latent, represent a primary hyperalgesia zone [37].

In Gifford’s mature organism model, exercise and manual therapies are thought to be most effective in patients with nociceptive pain, indicating that abnormal peripheral nerve input is the most important causative factor [38]. From a clinical viewpoint, in patients with the nociceptive phenotype, functional activity and early treatments that target the peripheral noxious input can be effectively used. This hypothesis is supported by meta-analyses reporting that manual therapies [39] and exercise programs [40] are effective strategies for decreasing pain and sensitivity in patients with TrPs.

### 2.2. Neuropathic Pain Phenotype

According to the IASP, pain can be considered neuropathic when 1. a lesion or disease of the somatosensory nervous system (i.e., central or peripheral nervous system) is identified as a cause of the pain; 2. pain is limited to a ‘neuroanatomically plausible’ distribution of the system; and 3. pain is supported by clinical examination, laboratory findings, and/or imaging results [41]. Patients with myofascial pain are not likely to be classified as having predominantly neuropathic pain, but TrPs can be present in patients with neuropathic pain (i.e., mixed-pain phenotype). For instance, Sari et al. found that 50% of patients with cervical radiculopathy also exhibited active TrPs in the muscles innervated by the affected nerve roots, contributing to the patients’ symptoms [42]. Similar findings were reported by Adelmanesh et al. in individuals with lumbar radiculopathy [43]. That study found that 76.4% of patients exhibited TrPs in the ipsilateral gluteal muscles in the distribution of the affected nerves [43]. These authors proposed that cervical root compression, i.e., a neuropathic phenotype, would be considered as initiating and/or maintaining of the TrPs [43]. Furthermore, preliminary evidence shows that TrP injection can be beneficial in subjects with lumbosacral radiculopathy [44]. In such cases, patients with a neuropathic pain phenotype would also be exhibiting secondary TrPs, thus showing a mixed phenotype. A patient with myofascial TrP gluteal pain but without radiculopathy would not be classified with a neuropathic pain phenotype. The seven-step approach to differentiate between the three major pain phenotypes as proposed below would remain relevant in such mixed types of pain, especially to identify the predominant type of pain.

### 2.3. Nociplastic Pain Phenotype

According to the IASP definition of nociplastic pain, central sensitization (e.g., increased responsiveness of nociceptive pain neurons within the central nervous system to their normal or subthreshold afferent input [45]) is thought to be the main underlying mechanism [11]. Thus, hyperalgesia, allodynia, and pain referred to distant sites are all indicators of nociplastic pain.

It is important to note that muscular referred pain, the cardinal sign of TrP pain, is explained as a manifestation of central sensitization initiated by a peripheral phenomenon and amplified and maintained by an additional sympathetic activity facilitation, and by dysfunctional descending pain inhibition [46,47]. In this way, the anatomical substrate of referred pain can be understood to be in the spinal cord (central) and to be the result of activation of quiescent axonal connections from peripheral nerves activated by peripheral painful input (a phenomenon that can occur in seconds) to dorsal horn neurons [48,49]. In support of this theory, Kuan et al. found that TrPs are more effective in inducing neuroplastic changes in the dorsal horn than non-TrP areas in the periphery [50]. Accordingly, it seems clear that central sensitization is involved in the development of spreading pain, since larger referred pain areas in individuals with chronic pain are the consequence of greater central neural plasticity [49]. It has been proposed that the presence of multiple TrPs (spatial summation) or the presence of TrPs for long-lasting periods (temporal summation) sensitize spinal cord neurons and supra-spinal structures because they are effective peripheral nociceptors whose afferent input can produce an afferent nociceptive barrage into the central nervous system. However, this has been an area that requires further study in order to support or refute this hypothesis.

Xu et al. reported that intramuscular stimulation of latent TrPs decreased pressure pain thresholds up to 30 min after intramuscular stimulation in healthy individuals [33], but this seems to be a normal primary hyperalgesic response (rather than evidence suggesting a possible etiological role of TrPs in central sensitization). In addition, studies by Niddam et al. found that, stimulation of TrPs induced enhanced activity of brain areas associated with the pain experience, including the primary and secondary somatosensory cortex, the inferior parietal, and the mid-insula and anterior insula compared to healthy pain-free subjects [51,52]. As these brain areas are commonly activated in acute, nociceptive pain, these findings support the notion of TrP causing sensitization processes.

Srbely et al. also suggested that TrPs could result from neurogenic mechanisms secondary to central sensitization; however, no further confirmation of this theory has been published [53]. If a central phenomenon can cause TrPs, it would be reasonable to assume that active TrPs would not be present in healthy, pain-free subjects where central sensitization mechanisms are not present, but latent TrPs have been found in this population [34].

The finding that diffuse noxious inhibitory control mechanisms can be blocked in the short term by experimentally induced myalgia suggests that clinical myofascial pain likewise exhibits nociplastic features [47]. Similarly, amplified vasodilatation in the referred pain area of patients with gluteus minimus TrPs also suggests nociplastic pain [54]. Other central-nervous-system-derived symptoms, e.g., fatigue, sleep problems, memory loss, concentration loss, or psychological disturbances, are also found in patients with nociplastic pain conditions [55] and may also be seen in individuals with myofascial pain, even if they are not specific or sensitive indicators of the activation of central pain mechanisms. The degree of nociplastic pain in patients with myofascial pain has been shown to be inversely related to improvements from a peripherally based treatment [56]. Therefore, identifying patients with nociplastic myofascial pain is important in order to institute proper therapeutic measures. In conclusion, myofascial TrP pain is fundamentally a nociceptive pain condition, and should not be classified primarily as neuropathic pain, but can be classified as nociplastic pain when it has features consistent with central sensitization, and can be part of a predominantly neuropathic or nociplastic pain condition as a comorbid disorder. When myofascial TrP pain syndromes accompany primarily neuropathic or nociplastic pain conditions, the treatment must be directed at both the primary condition as well as the comorbid myofascial pain. This is precisely what Dr. Janet Travel meant when she talked about the need to identify and treat perpetuating factors that affected myofascial pain when the perpetuating factor meant a comorbid illness. This also means that it is not enough to treat the obvious myofascial cause of chronic pain. The clinician must be aware of the need to assess the patient for another comorbid, and perhaps primary, condition.

## 3. Clinical Criteria/Grading System for Pain Phenotyping of Myofascial Pain

This section uses the IASP criteria and clinical reasoning process for determining the role of predominantly nociceptive or nociplastic pain in patients with myofascial pain [11]. A single patient can fulfill criteria for more than one pain phenotype. Moreover, a patient with a nociceptive or neuropathic phenotype can evolve to display a nociplastic pain phenotype. Therefore, it can be useful to first determine which pain phenotype is predominant at a particular moment.

### 3.1. Step 1—Duration of Pain

The first requirement for chronic pain diagnosis is that the pain symptoms are present for at least 3 months. There are patients with myofascial pain due to TrPs who experience pain over months or years. This criterion is assessed by taking the patient’s history.

### 3.2. Step 2—Distribution of Pain

Nociceptive pain patterns are usually discrete and regional, although despite being regional, somatic pain can be perceived as dull, deep, and poorly localized as opposed to being superficial and precise like a pinprick. It is generally exacerbated with defined pain triggers such as specific movements and activities. TrPs by definition are localized painful areas, so that even if feeling as a dull, poorly localized pain, the patient may be able to identify an area of pain with one finger, and the examiner can usually identify a small locus of intense pain when palpating muscle and eliciting a distant referred pain pattern. The referred pain is neuroanatomically appropriate since it is based on the activation of quiescent (silent) neurons at the spinal cord and therefore follows known peripheral nerve innervation patterns. [35]. Thus, TrPs in a C5 innervated muscle most often refer pain in a C5 nerve distribution. Because of the branching and spread of afferent nerve fibers in the spinal cord, the referred pain pattern of such TrPs would be found in C4,5,6 innervated muscle and dermatomes. A priori, a patient suffering from myofascial pain syndrome due to TrPs in a muscle, in what would be called somatic pain, would exhibit a nociceptive phenotype. The role that the referred pain has in determining phenotype is discussed below. Recent studies report that referred pain elicited by active TrPs reproduces symptoms in several localized chronic pain conditions such as neck pain [57], upper thoracic pain [58], shoulder pain [59], lateral elbow pain [60], or patellofemoral pain [61]. Similarly, active TrPs have been also found after minimal knee surgery (post-meniscectomy) [62]. The localized or regional pain in these conditions is nociceptive pain.

A nociplastic pain pattern is more generalized or widespread [14] than is nociceptive pain, but yet can be found in certain musculoskeletal pain syndromes. Fibromyalgia is a pain condition which fulfills several criteria for nociplastic pain [63,64]. In fact, widespread pain experienced by these patients is a cardinal sign of fibromyalgia, although other symptoms are always present. Preliminary studies have shown that the widespread pain pattern identified in fibromyalgia can be reproduced by the referred pain elicited by multiple active TrPs [65,66]. Similarly, patients with chronic widespread pain commonly have symptomatic myofascial TrPs [67]. Based on these findings, some authors have questioned if fibromyalgia is a pain syndrome due to multiple TPs or if TrPs in fibromyalgia are a manifestation of central sensitization (see previous discussion) [68]. Similarly, other authors propose that myofascial pain due to TrPs and fibromyalgia are different conditions, but with overlapping pain patterns [65,69]. In line with this theory, Plaut suggests that it is possible that myofascial TrP pain and fibromyalgia syndrome represent two ends on the same clinical spectrum [70]. In light of the current nociplastic pain criteria, it seems reasonable to conclude that fibromyalgia is a predominantly nociplastic pain condition.

Accordingly, a careful assessment and interpretation of the patient’s pain pattern during clinical assessment is needed to identify changes and the evolution of the symptoms from localized to a more generalized and widespread pattern. In such a scenario, pain drawings could be used to standardize and optimize the clinical assessment of an individual’s pain distribution in a reliable and valid way [71]. In fact, the use of pain drawings in clinical studies revealed that the widespread pain patterns experienced by women with fibromyalgia were formed by multiple regional painful areas, e.g., neck pain, low back pain, knee pain, and shoulder pain, rather than being “totally generalized” [72,73].

### 3.3. Step 3—Determine Whether Nociceptive Pain Is Present

The next step is to identify if the pain can be entirely explained by nociceptive mechanisms [14]. Imaging techniques (e.g., ultrasonography or magnetic resonance imaging) could help to identify a specific pain generator able to evoke nociception, which should be consistent with the patient’s self-reported pain pattern. Since manual TrP diagnostic paradigms have an inherent subjectivity, recent advancements in imaging [74] and TrP-specific biomarkers [75] have been utilized to provide objective indicators that could improve the identification of myofascial TrPs. Imaging studies have visualized muscle taut bands and identified that taut bands harboring TrPs exhibit greater stiffness and also reduced vibration amplitude when compared with normal muscle sites [27,28,74].

It is important to note that identification of a source of nociception does not exclude the possibility of concomitant nociplastic or neuropathic pain. Since evidence supports that muscle TrPs represent a peripheral source of nociception and thereby are drivers for the development of sensitization, it is possible that long-lasting and even short- to moderate-term myofascial pain from nociceptive generators could facilitate central sensitization and, therefore, evolve to a nociplastic phenotype. TrPs indeed have been found to be associated with widespread pressure pain sensitivity in such painful conditions as tension-type headaches [76], chronic neck pain [77], and head/neck cancer pain [78].

### 3.4. Step 4—Determine Whether Neuropathic Pain Is Present

Another mandatory criterion for nociplastic pain is that symptoms cannot be explained in their entirety by neuropathic pain mechanisms [14]. As previously stated, a patient with myofascial TrP pain is unlikely to show neuropathic signs unless there is a comorbid neurological disorder. However, it is possible that a patient with a neuropathic pain condition will also have myofascial pain, as has been observed in patients with cervical [42] or lumbar radiculopathy [43]. Involvement of the nervous system may lead to sensitization of muscles, causing the development of TrPs in those nerve-related muscles [42,43]. This situation would lead to a mixed-type pain phenotype, e.g., neuropathic and nociceptive phenotypes together.

Similarly, there is a great deal of overlap between neuropathic and nociplastic phenotypes. Indeed, sustained neuropathic pain also results in increased hyper-excitability of the central nervous system [79]. As an example, fibromyalgia syndrome is also considered a neuropathic condition as well as a nociplastic condition by different authors [80]. The progression from a neuropathic pain phenotype to a nociplastic pain phenotype provides one explanation for the spreading of the pain beyond the distribution of the affected nerves.

### 3.5. Step 5—Elucidate the Presence of Pain Hypersensitivity

The fifth step involves screening for signs of pain hypersensitivity, including hyperalgesia (defined as an exaggerated pain response to painful stimuli) and allodynia (defined as pain in response to stimuli that normally do not elicit pain) responses [14]. An important differentiation is that the nociceptive phenotype is characterized by primary hyperalgesia (i.e., hypersensitivity in the painful/affected region and appropriate to the pain-inducing stimulus), whereas nociplastic pain is characterized by secondary hyperalgesia (i.e., hypersensitivity within and outside the painful/affected region and by a greater than expected perception of pain in response to a given stimulus). Several studies have shown that myofascial pain mainly exhibits pressure pain hyperalgesia in the affected area [20,21,22,23,24,25]. Hyperalgesic and allodynic responses can be present in distant pain-free areas in some individuals. According to the IASP clinical criteria, if the first five steps are positive for nociplastic pain, a patient can be classified as having “possible nociplastic pain” [14].

### 3.6. Step 6—Check for the History of Pain Hypersensitivity

Step 6 involves examining whether the patient reports symptoms of pain hypersensitivity, particularly allodynic responses, with activities of daily living, e.g., wearing a belt or wearing jewelry, during bathing or showering, or from sitting due to pressure on the buttocks. If the patient meets criteria in step 6, then pain can be considered “probably nociplastic pain” [14]. Allodynia can be present in some patients with myofascial pain, though it is not common.

### 3.7. Step 7—Determine Whether Comorbidities Are Present

The final step involves screening for sensitivity to other stimuli, including sensitivity to sound (phonophobia), light (photophobia), and to the presence of comorbid medical conditions, as well as central nervous system-associated symptoms such as poor sleep quality, fatigue, and cognitive problems [14]. It is important to emphasize that even though myofascial TrPs can be a primary dysfunction not associated with any medical condition, and would therefore fulfil the nociceptive pain phenotype criteria, they can also be comorbid with other medical conditions associated with central sensitization, such as painful knee osteoarthritis [81], migraine [82], cancer, [83], and dysmenorrhea [84]. For example, TrP activation due to visceral nociception may persist after appropriate treatment due to nociceptive input from TrPs located in the referred visceral area related to centrally induced visceral hyperalgesia [85]. Accordingly, in those individuals where TrPs are comorbid to an established medical pathology, the presence of nociplastic pain is more plausible. Clinicians should differentiate TrP-related pain as a primary nociceptive cause of symptoms, e.g., pelvic floor muscle myofascial pain, from referred pain to the pelvic girdle from primary visceral pain syndromes such as painful bladder, cytistis, or endometriosis, producing comorbid myofascial pain, as well as referred pain from the somatic body wall muscles that mimic visceral organ pain [86]. Predominantly nociplastic pain can be associated with comorbid TrPs, as in the above examples, implying that the underlying nociplastic pain mechanisms (i.e., peripheral and/or central sensitization) are primarily driving the clinical picture, rather than the secondary nociceptive mechanisms (i.e., TrPs).

**Figure 1:**
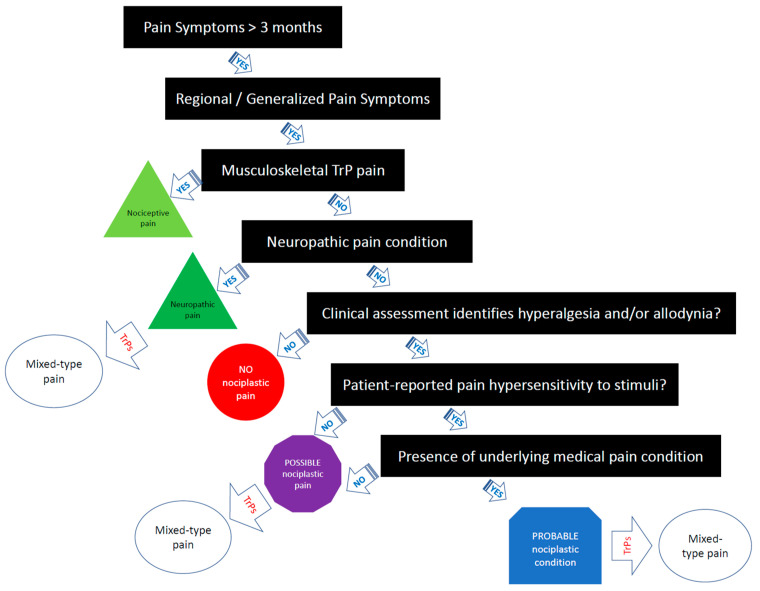
Clinical decision-making tree of the IASP clinical criteria for myofascial TrP pain.

If all criteria are met, the comorbid myofascial TrP pain should be classified as “probable nociplastic pain” [14]. Figure 1 provides a clinical decision-making tree for clinicians based on the IASP clinical criteria for assessing pain phenotype in people with myofascial TrP pain. It is important to note that more research is needed to examine the reliability and validity of the 2021 IASP clinical grading criteria [14]. Additionally, more research is needed to determine the prognostic value and utility of the IASP clinical criteria on treatment outcomes in clinical trials.

## 4. Considering Pain Phenotype into Myofascial Trigger Point Treatment

The challenge facing clinicians treating myofascial pain syndrome is always to determine the proper treatment approach for each individual patient, as patients are likely to be different in their clinical presentations and their treatment response may depend on their pain phenotype. Thus, the application of the IASP clinical criteria [14] to individuals with myofascial TrP pain would allow clinicians to provide more precise and appropriate treatment strategies according to the pain phenotype. Accordingly, when developing a treatment plan, determining the predominance of a nociceptive or nociplastic pain phenotype should be included in the clinical decision tree. The absence of this reasoning process could explain some of the discrepancies in current treatment guidelines for myofascial TrP pain syndrome.

Current management approaches for TrP pain are mainly focused on peripheral strategies targeted to inactivate the TrP [87]. Meta-analyses support the use of manual therapies [39] and dry needling [88] for decreasing pain and sensitivity in the short term in people with TrP pain. However, evidence shows that the effects of these interventions have mostly short- or moderate-term benefit but minimal long-term benefit [39,88]. A potential explanation of the lack of long-term benefit following local TrP treatment could be related to the lack of identification of those individuals with a nociplastic phenotype requiring a more extensive or holistic treatment approach. Integrating TrP treatment strategies into contemporary pain neurosciences when providing TrP treatments, particularly dry needling, has been previously proposed [89]; however, no clinical decision tree identifying the predominant myofascial TrP pain phenotype as nociceptive, nociplastic, or mixed has been developed.

Current evidence supports that TrPs can be present in predominantly nociplastic conditions such as fibromyalgia. Accordingly, treatments directed at TrPs should be only considered as part of a more extensive treatment program that is primarily directed at the underlying nociplastic pain mechanism. In fact, evidence suggests that primarily treating the peripheral generators (e.g., myofascial TrP) in a nociplastic pain condition (e.g., in fibromyalgia patients) results in a lesser degree of diffuse pain due to central sensitization, thus potentially reducing the necessity for specific pain treatments [90,91]. The latter includes treatment targeting those factors which perpetuate and interact with pain (e.g., sleep disturbances, anxiety, kinesiophobia, and depression). Accordingly, in a patient with mixed nociceptive and nociplastic pain without a clear predominantly nociplastic state, the treatment plan should be multimodal, including management of perpetuating and predisposing TrP-inducing factors, since removing the peripheral nociceptive input from TrPs could potentially have the effect of partially modulating the central nervous system pathways. This approach should include pharmacological treatment, physical therapy (also including dry needling [92,93]), as well as psychological or cognitive behavioral approaches, e.g., pain neuroscience education. Therefore, multimodal treatment approaches integrating a biopsychosocial model, which address relevant comorbidities and lifestyle factors for each patient, might further improve the outcome for patients with the myofascial nociplastic pain phenotype. A major aim of treatment of individuals with nociplastic pain is that they will develop different strategies to optimize functional movement and undertake active exercise.

Mata Diz et al. found that combining stretching and strengthening was effective over the short term for decreasing TrP pain [94]. Ahmed et al. concluded that aerobic exercise is a potentially effective treatment for myofascial TrP pain [95]. However, we do not currently know which type of exercise, e.g., localized or aerobic, resistance or strengthening, is better for the management of myofascial TrPs [41]. The underlying pain phenotype should be determined in order to optimize the exercise prescription, especially in people with a nociplastic pain phenotype [96]. In patients with a nociplastic phenotype, exercise should be applied/graded in a pain-contingent way [97], and pacing or cognitive approaches should be applied either in isolation or combined with exercise. In subjects with a nociceptive phenotype, exercise could be more “pain” provocative since painful exercises offer significant benefit over pain-free exercises, at least in the short term (moderate quality evidence) [98]. A potential reason that could explain the positive effect of painful exercise is that a brief painful stimulus is insufficient to induce or maintain central nervous system sensitization since the response within the pain neuromatrix is strongly influenced by the context in which the painful stimulus appears, especially within the context of a therapeutic encounter [99].

Based on the proposed reasoning, treatment of the nociceptive phenotype will be based mostly on tissue-focused interventions, e.g., bottom-up techniques, whereas management of the nociplastic pain phenotype would also include central nervous system interventions, e.g., top-down techniques [100]. This proposal should be investigated in future research.

## 5. Conclusions

Myofascial pain has been traditionally classified as nociceptive pain. The current paper summarizes data supporting the of subgrouping of patients with myofascial TrP pain into nociceptive, nociplastic, or mixed-type phenotype. Simple myofascial pain syndrome caused by TrPs is primarily a nociceptive condition, is unlikely to be classified as neuropathic or nociplastic, but can be present in patients as a comorbid condition along with predominantly neuropathic or nociplastic pain syndromes. The presence of underlying painful nociplastic comorbidities may lower the threshold for pain by increasing the central nervous system gain, leading to sensitization, and hence TrPs may become a painful generator of pain. In the latter cases, treatment of a predominantly central pain problem tends to be the primary management goal, but the peripheral drive from myofascial pain should not be ignored since TrP treatment has been shown to reduce sensitization-associated symptomatology in nociplastic pain, e.g., fibromyalgia. We propose a clinical decision tree by using the 2021 IASP classification grade criteria for identifying the predominant pain phenotype in people with myofascial TrP pain. Nociplastic pain is considered to be more difficult to treat than nociceptive pain alone and requires a more nuanced, multimodal treatment approach to achieve better treatment outcomes.

## Data Availability

Not applicable.
